# A System of Obstetric Medicine and Surgery, for the Student and Practitioner

**Published:** 1885-12

**Authors:** 


**A System of Obstetric Medicine and Surgery, Theoretical and Clinical, for the Student and Practitioner.** By ROBERT BARNES, M. D., and FANCOURT BARNES, M. D. Illustrated with two hundred and thirty-one wood cuts. Philadelphia: Lea Brothers & Co. 1885.

The publication of this work, from the pen of Robert Barnes, is a most important contribution to the literature of obstetrics. For many years, the author has given the profession valuable original papers on obstetrics and allied subjects, and it may be safely stated that no other writer has wielded an equal influence. A considerable part of the field, covered in the "Obstetric Operations," a work which placed the author in the front rank as a writer on obstetric subjects, is included in the present volume, and he has also incorporated the latest advances in the practice of difficult midwifery. With the assistance of his son, Fancourt Barnes, the ripe experience of the distinguished author, and his wide and extended study of obstetrics in all its departments, are here preserved and presented to the profession.

The work is profusely illustrated. It presents the clearest study of the mechanism of labor of any work in the English language. The chapters devoted to puerperal diseases are full, and furnish the latest and best opinions upon these most important pathological conditions.

In a careful review of the work, we are impressed with its value to the practitioner. It is full and complete, and presents the subject in a logical manner. It compels the student to study closely in order to comprehend the solid arguments embodied in the various subjects treated. As a whole, therefore, we cannot commend the work too highly. It is what we expected it would be when noticing the announcement of its publication—the most comprehensive treatise on obstetrics published in the English language.

